# A deep‐sea bacterium related to coastal marine pathogens

**DOI:** 10.1111/1462-2920.15629

**Published:** 2021-06-15

**Authors:** Aide Lasa, Manon Auguste, Alberto Lema, Caterina Oliveri, Alessio Borello, Elisa Taviani, Guido Bonello, Lapo Doni, Andrew D. Millard, Maxime Bruto, Jesus L. Romalde, Michail Yakimov, Teresa Balbi, Carla Pruzzo, Laura Canesi, Luigi Vezzulli

**Affiliations:** ^1^ Department of Earth, Environmental and Life Sciences (DISTAV) University of Genoa Genoa Corso Europa 26, 16132 Italy; ^2^ Department of Microbiology and Parasitology CIBUS‐Facultade de Bioloxía & Institute CRETUS, Universidade de Santiago de Compostela Santiago de Compostela 15782 Spain; ^3^ Department of Genetics and Genome Biology University of Leicester University Road, Leicester UK; ^4^ Sorbonne Universités, UPMC Paris 06, CNRS, UMR 8227, Integrative Biology of Marine Models, Station Biologique de Roscoff CS 90074 Roscoff Cedex F‐29688 France; ^5^ Institute of Biological Resources and Marine Biotechnology, National Research Council (IRBIM‐CNR) Messina 98122 Italy

## Abstract

Evolution of virulence traits from adaptation to environmental niches other than the host is probably a common feature of marine microbial pathogens, whose knowledge might be crucial to understand their emergence and pathogenetic potential. Here, we report genome sequence analysis of a novel marine bacterial species, *Vibrio bathopelagicus* sp. nov., isolated from warm bathypelagic waters (3309 m depth) of the Mediterranean Sea. Interestingly, *V. bathopelagicus* sp. nov. is closely related to coastal *Vibrio* strains pathogenic to marine bivalves. *V. bathopelagicus* sp. nov. genome encodes genes involved in environmental adaptation to the deep‐sea but also in virulence, such as the R5.7 element, MARTX toxin cluster, Type VI secretion system and zinc‐metalloprotease, previously associated with *Vibrio* infections in farmed oysters. The results of functional *in vitro* assays on immunocytes (haemocytes) of the Mediterranean mussel *Mytilus galloprovincialis* and the Pacific oyster *Crassostrea gigas,* and of the early larval development assay in *Mytilus* support strong toxicity of *V. bathopelagicus* sp. nov. towards bivalves. *V. bathopelagicus* sp. nov., isolated from a remote Mediterranean bathypelagic site, is an example of a planktonic marine bacterium with genotypic and phenotypic traits associated with animal pathogenicity, which might have played an evolutionary role in the origin of coastal marine pathogens.

## Introduction

Understanding the emergence of bacterial pathogens as well as the origin and evolution of their pathogenicity potential is of great importance for the comprehension of infectious diseases epidemiology affecting humans and animals. It has been long believed that the complex interactions occurring between pathogens and the infected hosts are the primary driving forces that determine the strategies used by microorganisms to counter host defence. However, new evidence suggests that the external (non‐host) environment might play a greater role in the evolution of certain pathogens and their virulence traits than previously thought (Nakagawa *et al*., 2007; Vezzulli *et al*., 2008; Hasan *et al*., 2015). This holds particularly true for non‐obligatory parasite that spend a substantial part of their life cycle outside hosts, but once introduced into the host cause disease with measurable frequency (Gerba, 2015).

The naturally occurring gram‐negative bacteria belonging to the genus *Vibrio* comprise several species pathogenic to humans and animals and are widespread in the marine environment. They are more common in warmer coastal waters, especially above 17°C, and depending on the species, they tolerate a range of salinities (5–25 ppt; Ceccarelli *et al*., 2019). Several *Vibrio* species have been associated with diseases in marine invertebrates (Wilson *et al*., 2013), including oyster spat and/or larvae (Destoumieux‐Garzón *et al*., 2020), and are associated with mortality outbreaks affecting the production of the Pacific oyster *Crassostrea gigas* worldwide (Lemire *et al*., 2015; Vezzulli *et al*., 2015; Bruto *et al*., 2017, 2018; Rubio *et al*., 2019; Oyanedel *et al*., 2020). In particular, *Vibrio* species belonging to the Splendidus clade have been repeatedly isolated from oysters suffering the ‘summer mortality syndrome’ and experimentally showed to cause death when injected to bivalves (Le Roux *et al*., 2007). A number of virulence factors have been reported to cause disease, including cytolisin and secreted metalloproteases, many of them shared by phylogenetically coherent virulent population (Lemire *et al*., 2015; Bruto *et al*., 2017). Interestingly, it was recently reported that virulence potential in *Vibrio* populations may derive from the acquisition of ancestral genes such as the case of the R5.7 exported conserved protein within the Splendidus clade and the MARTX toxin cluster in *Vibrio splendidus* (Bruto *et al*., 2018; Oyanedel *et al*., 2020). According to the coincidental selection hypothesis factors responsible for virulence may have resulted from adaptation to other ecological niches other than the host. Coincidental selection is well exemplified by *Vibrio cholerae* virulence factors involved in resistance to protozoan grazing in the marine environment (Van der Henst *et al*., 2018). Studies on pathogenicity of environmental vibrios and other related microbial pathogens also provided evidence that some virulence factors (also named ‘Dual Role Virulence Factors‐DRVFs’) used by pathogens during human and animal infection may have primary evolved for survival in the aquatic habitat and later coincidentally adapted to host infection (Nakagawa *et al*., 2007; Vezzulli *et al*., 2008). The existence of DRVFs suggests that the ability to use the same structure(s) to interact with different substrates (e.g., in the environment and in the host) may be a common property of pathogenic bacteria having environmental reservoirs and that this may represent a discriminating feature between the harmless and the potentially pathogenic environmental bacteria. Ultimately, DRVFs may also represent good targets for developing novel prophylactic or therapeutic interventions that not only affect the success of an infection (e.g., pathogen–host interaction) but also the ecological fitness of the microbial pathogen in the non‐host environment (Vezzulli *et al*., 2008).

Deep‐Sea Mediterranean basins, dating to about five million years ago, are remote, pristine and stable environments offering a peculiar evolutionary context for microbes, well separated to conditions found in coastal marine areas (Martın‐Cuadrado *et al*., 2007). Notably, deep Mediterranean water mass never gets below 13.5°C, representing a unique relatively warm deep habitat suitable for the isolation of vibrios, thus offering a great opportunity to study the origin and evolution of environmental microbial pathogens and the identification of new DRVFs.

In this study, we report the isolation, characterization and full genome sequence analysis of a novel *Vibrio* species, *Vibrio bathopelagicus* sp. nov., isolated from warm bathypelagic Mediterranean waters, which is phylogenetically closed and shows genomic virulence traits similar to those found in coastal *Vibrio* strains pathogenic to bivalves. The results of in vitro challenge assays carried out in immune cells (haemocytes) of the Mediterranean mussel *Mytilus galloprovincialis* and the Pacific oyster *C. gigas* and of the 48 h larval toxicity assay in mussels support toxicity of this species towards bivalves. *Vibrio bathopelagicus* sp. nov. provides evidence on roots of bacterial virulence that may have contributed to the emergence and evolution of coastal bacterial strains pathogenic for bivalves.

## Results and discussion

### Strain identification and taxonomy

A Gram‐negative, motile and bacillar shape bacteria was isolated from the Ionian station Sal10^T^ in the Mediterranean Sea at the depth of 3309 m (Figure S1). The strain was sucrose negative in thiosulfate‐citrate‐bile salts‐sucrose agar (TCBS) media, and oxidase and catalase positive. Growth in the presence of NaCl was observed in the range of 1–50 ‰ and in the presence of sea salts from 1 to 45 ‰. The isolate grew well in a wide range of pH (3–12), and growth was observed at temperatures in the range of 4–30°C.

Identification of the isolate was preliminary performed through the analysis of 16S rRNA gene sequences which were retrieved from the genome, resulting in a total of 15 sequences, 14 in chromosome I (C‐I) and 1 in chromosome II (C‐II). It is well known that the number of rRNA operons varies among bacterial genomes from 1 to 15 copies (Pei *et al*., 2010) and over 80% bacterial genomes sequenced possess more than one operon. Multiple rRNA copies would result in a selective pressure to maintain and rapidly increase high ribosome content that would enable rapid adaptation to nutritional upshift or favourable temperature change (Klappenbach *et al*., 2000; Roller *et al*., 2016), although multiple rRNA operons are not essential. Accordingly, deep sea bacteria are known to harbour a high ratio of rRNA operon copies per genome (Lauro and Bartlett, 2008).

**Fig. 1:**
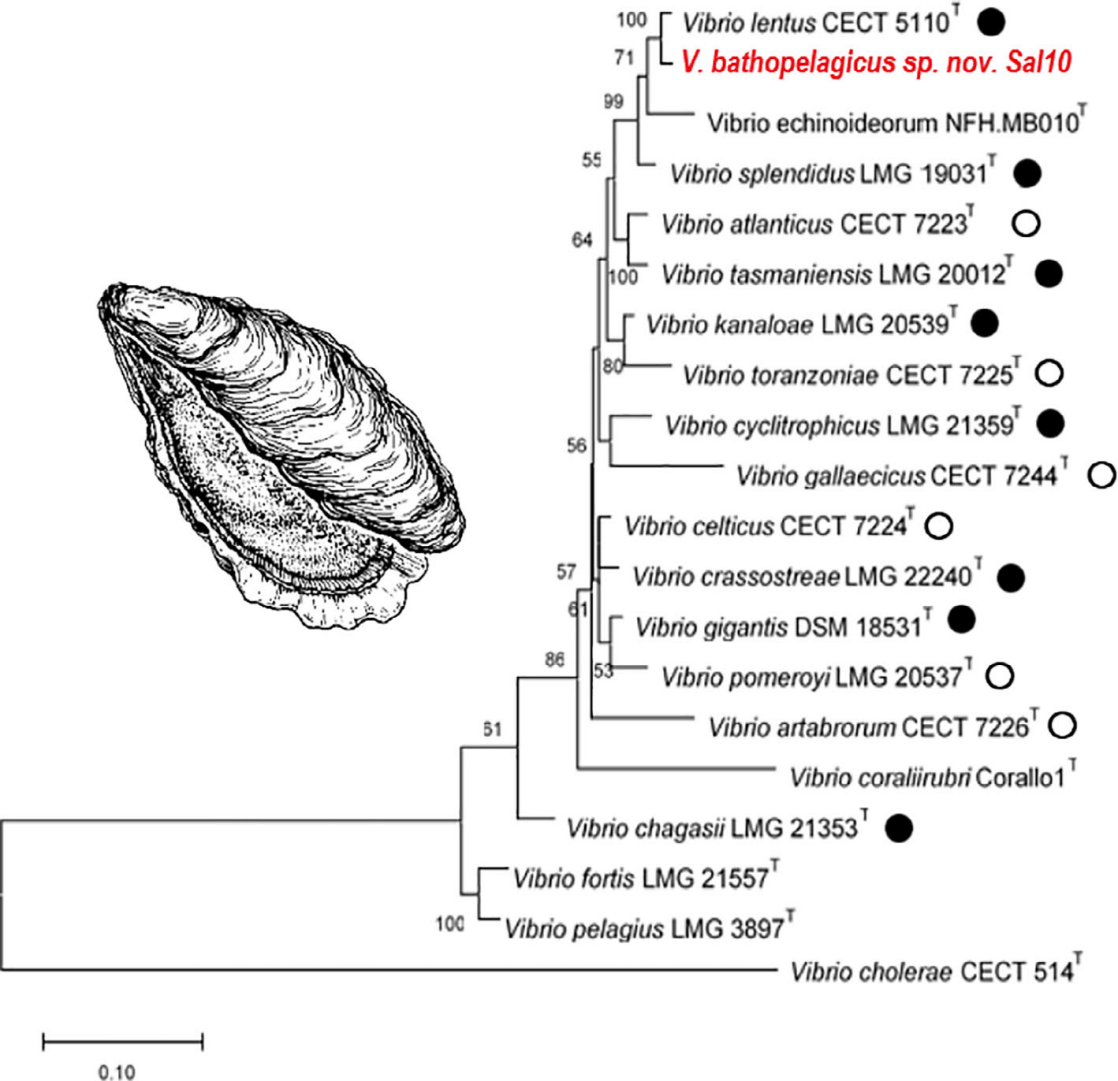
Maximum‐Likelihood (GTR+G+I parameters) phylogenetic tree based on concatenated sequences of *atpA*, *pyrH*, *recA*, *rpoA* and *rpoD* genes. Only bootstrap values (1000 replications) above 50% are shown. *Vibrio cholerae* CECT 514^T^ = ATCC 14035^T^ was used as an outgroup. Black circles: *Vibrio* species found in association with oysters; white circles: *Vibrio* species found in association with other marine bivalves (Lemire *et al*., 2015; Destoumieux‐Garzón *et al*., 2020).

Phylogenetic analysis of 16S rRNA gene combined with multilocus sequence analysis (MLSA), including five housekeeping genes (*atp*A, *pyr*H, *rec*A, *rpo*A and *rpo*D) showed that Sal10^T^ strain branched with the Splendidus clade species (Figure 1). This result was confirmed when a RAxML phylogenetic tree was constructed based on 100 housekeeping genes obtained from all the Splendidus clade species genomes present in the databases (Figure S2). The evolutionary tree fully resolved the phylogeny, placing Sal10^T^ and *Vibrio lentus* in a monophyletic branch that corroborated the results of the MLSA based on five housekeeping genes. Species delineation was determined by Average Nucleotide Identity (OrthoANI) and *in silico* DNA‐DNA hybridization (*is*DDH) between Sal10^T^ isolate and the closest relatives, with highest OrthoANI and *is*DDH values with *V. lentus*, 91.16% and 43.70% respectively, both of which are under the species delineation threshold values. Sal10^T^ strain can be differentiated from its closest relatives by several phenotypic features, such as the inability to hydrolyse arginine, to grow at 6% NaCl, acetoin production (Voges–Proskauer reaction), and utilization of glucuronic acid, maltose, d‐galactose and aesculin hydrolysis (Table S1). Together, these findings confirm separate species demarcation for Sal10^T^ strain for which the species name *V. bathopelagicus* sp. nov. is proposed.

### Description of *V. bathopelagicus* sp. nov. (ba.tho.pe. la'gi.cus. Gr. adj. bathos deep; L. masc. adj. pelagicus, from the sea; N.L. masc. adj. bathpelagicus, belonging to the deep sea)

Cells are Gram‐stain‐negative, rod‐shaped and motile. Colonies were circular with irregular edges and cream coloured in MA medium after 24 h at 24°C. The strain was sucrose negative in TCBS, oxidase and catalase positive, and susceptible to O/129 (10 μg and 150 μg per disc). Bacteria grow at 4–30°C and in the presence of 1‐5% (w/v) NaCl and in the presence of 1–4.5% of Sea Salts (wt/vol). The isolate grew well from pH 3 to 12. Nitrates are reduced to nitrites. Indole is not produced and hydrolysis of esculin, gelatine and starch are observed. Decarboxylation of arginine, lysine and ornithine is not produced, urease reaction is negative, whereas citrate and gas glucose are positive. Enzymatic activity is observed for alkaline phosphatase, esterase lipase (C8), leucine arylamidase, valine arylamidase, trypsin, acid phosphatase, Naphthol‐AS‐BI‐phosphohydrolase and *N*‐acetyl‐beta‐glucosaminidase, but no activity for esterase (C4), lipase (C8), cystine arylamidase, alpha‐chymotrypsin, alpha‐galactosidase, β‐galactosidase, beta‐glucuronidase, alpha‐glucosidase, beta‐glucosidase, alpha‐mannosidase and alpha‐fucosidase. Use as sole carbon source of citric acid, fumaric acid, glucuronic acid, malic acid, pyruvic acid, succinic acid, sodium acetate, d‐galactose, d‐gluconate, glycerol, glycine, glucose, histamine, maltose, mannitol, ribose, sucrose, serine, tyrosine and threonine are positive, while use of alpha‐ketoglutaric acid, galacturonic acid, glutamic acid, hydroxybutyric acid, propionic acid, saccharic acid, trans‐aconitic acid, amygdaline, arabinose, citrulline, phenylacetate, phenylalanine, lactose, leucine, lysine, melibiose, myo‐inositol, *N*‐acetyl glucosamine, ornithine, salicine, sarcosine and xylose are is observed.

The type strain, Sal10^T^ (= CECT30197^T^ = LMG 32069^T^), was isolated from the water column of Sal10 station in the Ionian Sea at a depth of 3309 m. The DNA G+C content (genome) of the type strain is 44.05 mol%. The assembled genome sequence of strain Sal10^T^ is deposited at DDBJ/ENA/GenBank under the accession CP062500‐CP062501 and 16S rRNA consensus sequence under the accession MW195017.

### Genomic features

**Fig. 2:**
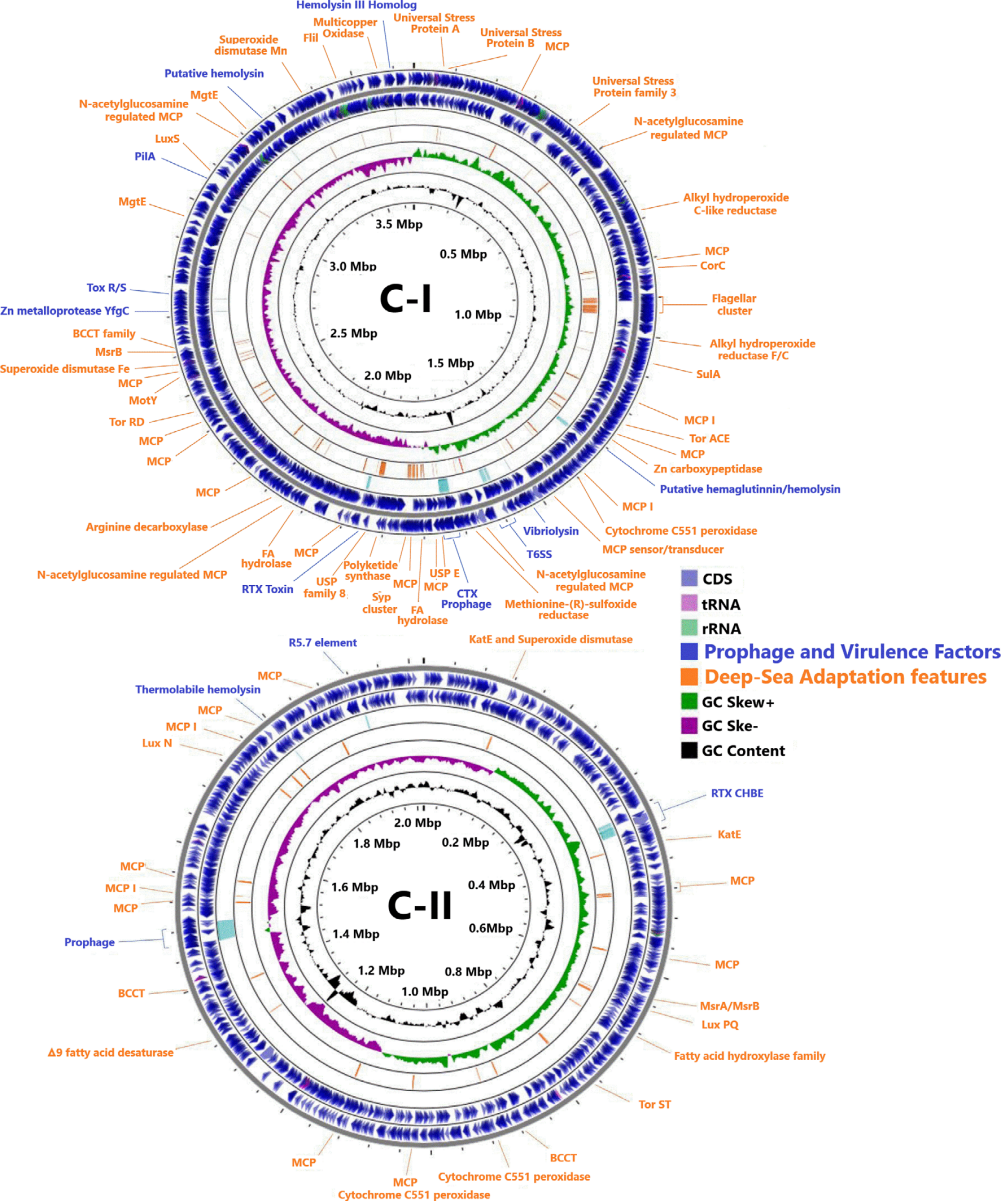
Full genome map of Sal10^T^ indicating the adaptation features (orange colour), putative virulence factors and prophage (blue colour) position on each chromosome.

Completion of Sal10^T^ genome was achieved by combining different sequencing technologies and obtaining a hybrid assembly that comprised two chromosomes (Figure 2, Table S2). The larger chromosome (C‐I) was 3 649 238 bp in length with a 44.18% GC content, while smaller chromosome (C‐II) was 2 018 969 bp in length and a 43.92% GC content. Annotation of both chromosomes detected 3 255 genes (2 589 coding sequences with functional assignment) and 161 RNAs (118 tRNAs and 43 rRNAs) in C‐I, whilst C‐II harboured 1 797 genes (1 271 coding sequences with functional assignment) and 19 RNAs (16 tRNAs and 3 rRNAs). Gene content distribution across chromosomes displayed a similar pattern to that observed in other Vibrios, with C‐I containing mainly genes associated to viability and growth and C‐II including genes related to environmental adaptation. The Sal10^T^ genome encoded a total of 50 genomic islands (GIs) including 627 genes, 45 GIs were allocated in C‐I and 5 GIs in C‐II. Genes related to toxins, modification‐restriction systems, antibiotic resistance, LPS modification, metabolism, and other putative proteins with unknown functions were found in the GIs. For example, a two‐component regulatory system PhoP/PhoQ was present in C‐I, known to regulate the expression of genes involved in virulence, adaptation to acidic and low Mg^2+^ environments and resistance to host defence antimicrobial peptides, or a leukotoxin that plays an important role in immune evasion. Genes encoding biosynthetic pathways responsible for the production of secondary metabolites were found in C‐I and six different gene clusters of secondary metabolites synthesis were predicted by antiSMASH and summarized in Table S3. The assembled completed genome sequence of strain Sal10^T^ is deposited at DDBJ/ENA/GenBank under the accession CP062500‐CP062501 and 16S rRNA consensus sequence under the accession MW195017.

### Deep‐sea adaptation

The genome of Sal10^T^ harboured a number of genes that were previously predicted to protect bacteria under the extreme conditions of the deep‐sea environment that are summarized in Table 1. (Vezzi *et al*., 2005; Goudenège *et al*., 2014; Hasan *et al*., 2015) Functions annotated in the genome included different systems of protection against O_2_ reactive species, such as Cytochrome C_551_ peroxidase or KatE catalase, both detoxify H_2_O_2_, an alkyl hydroperoxide reductase that scavenge endogenous hydrogen peroxide and genes encoding different superoxide dismutase for tolerating high O_2_ concentrations. These genes have been previously found in *Vibrio antiquarius* EX25 a deep‐sea hydrothermal vent strain (Hasan *et al*., 2015). Methionine‐(R)‐sulfoxide reductase (MrsA and MrsB) have been shown to play an important role in response to oxidative stress by repairing methionine residues oxidation preventing protein oxidative damage (Singh *et al*., 2018).

**Table 1:** Predicted deep‐sea adaptation characteristics of Sal10T genome

Predicted biology	Chromosome (occurrence)	Function
*Response to environment*		
Cytochrome c551 peroxidase	C‐I, C‐II (2)	Protection against O_2_ and H_2_O_2_
Catalase KatE	C‐II (2)	
Superoxide dismutase (Fe), (Mn), (Cu‐Zn)	C‐I (2), C‐II	
Alkyl hydroperoxide reductase	C‐I	Scavenge endogenous hydrogen peroxide
Methionine‐(R)‐sulfoxide reductase (MrsA, MrsB, free reductase)	C‐I (2), C‐II	Oxidative damage repair
Trimethylamine‐*N*‐oxide reductase system torA,C,D,E,R	C‐I	TMAO respiration involved in High Hydrostatic Pressure adaptation
Trimethylamine‐*N*‐oxide reductase system S,T	C‐II	
∆‐9 fatty acid desaturase	C‐II	Fatty acid unsaturation, essential for growth under high pressure
Other fatty acid desaturases	C‐I (2), C‐II	
Polyketide synthase	C‐I (2)	Synthesis of polyunsaturated fatty acids
Zinc carboxypeptidase	C‐I	Functioning in high concentrations of HM
Magnesium transporters (MgeT, CorC)	C‐I	Growth at high MgCl_2_ concentrations
Betaine‐choline‐carnitine transporter (BCCT) family	C‐I, C‐II (2)	Acquisition of different osmoprotectants
*Cell sensing system*		
Lux S	C‐I	Autoinducer‐2 (AI‐2) mediated QS, biofilm formation, virulence, other metabolic functions
Lux P,Q,N	C‐II	
Methyl‐accepting chemotaxis proteins (MCPs)	C‐I, C‐II	Signal transducing proteins, respond to gradients of chemicals in the environment. Maximize productivity and growth in low nutrients environments
*Biofilm‐related pathways*		
Arginine decarboxylase	C‐I	Polyamine biosynthesis
c‐di‐GMP phosphodiesterase mbaA	C‐I, C‐II	Norspermidine
Syp gene cluster	C‐I	Gene clusters mediating biofilm formation
Flagellar cluster	C‐I	
*Others*		
rmf and sulA	C‐I	Persister cells
Multicopper oxidase	C‐I	Mn(II) oxidation
Universal stress protein family	C‐I	

High hydrostatic pressure (HHP) adaptation involves different metabolic systems, such as Trimethylamine‐*N*‐oxide (TMAO) reductase system that is present in Sal10^T^ genome (Table 1). TMAO can be produced through oxidation of TMA by a variety of marine bacteria, including *Vibrio fluvialis*, *V. cholerae*, *Photobacterium phosphoreum* or different deep‐sea *Shewanella* species (Wang *et al*., 2008; Aono *et al*., 2010; Zhang *et al*., 2016), and serves to protect against osmotic stress, adverse effects of low temperature, high concentration of urea or HHP but also as an electron acceptor of anaerobic respiration. It has been suggested that TMAO may provide the bacterial cell an alternative source of energy when oxygen concentration decreases and HHP induces the expression of TMAO reductase system.

Genome of Sal10^T^ strain also contains genes for fatty acid unsaturation synthesis, including ∆−9 fatty acid desaturase or polyketide synthase, which are essential for growth under HHP by increasing membrane fluidity (Lauro and Bartlett, 2008). Two different classes of Mg^2+^ transporters have been identified, MgeT and CorC (Table 1), responsible for the maintenance of a correct Mg^2+^ homeostasis for fundamental cell functioning. Additionally, betaine‐choline‐carnitine transporter genes family were detected. Many halophilic bacteria adjust their cell turgor pressure through the acquisition of different osmoprotectants from the surroundings, such as ectoine, betaine, carnitine or choline, or by the synthesis from their precursors (Oren, 2008; Zeaiter *et al*., 2019). The presence of these transporters may contribute to the accumulation of these osmoprotectants inside the bacterial cell favouring the adaptation to high hydrostatic pressure.

Sensing of environmental cues represents a key factor that favours microbe survival and allows them to move away from toxic compounds. Methyl‐accepting chemotaxis proteins (MCPs) are the main chemoreceptors in bacteria involved in regulation of diverse aspects of cellular activities including biofilm formation, flagellum biosynthesis, degradation of xenobiotic compounds, encystment and fruiting body formation, exopolysaccharide and toxins production and pathogenicity (Berleman and Bauer, 2005; Hickman *et al*., 2005; Kirby, 2009; Nishiyama *et al*., 2016). The hunt for dissolved and particulate organic matter makes MCPs proteins (usually found in high number) a peculiar genomic feature characterising bacterial strains indigenous to hadal and abyssal environments (Lauro and Bartlett, 2008). Accordingly, MCPs has been detected in large number in the genome of Sal10^T^ strain (Figure 2).

Other quorum sensing and biofilm formation systems (*Lux* operon) and flagellar cluster present in the deep Sal10T bathytype, may reflect its ability to survive and adapt under harsh environmental conditions. Response to small concentrations of nutrients may also led the bacteria into a low metabolic activity by forming persister cells promoting cell downsizing, thus reducing cell surface, mediated by different systems including autoinducer‐2 (AI‐2), ribosome modulation factor (*rmf*) and cell division inhibitor (*sulA)* genes (Hasan *et al*., 2015; Song and Wood, 2020). Interestingly, the photolyase gene (*phr*), involved in cyclobutene pyrimidine dimers repair in UV irradiated DNA, has been found in Sal10^T^ strain, as well as in several deep‐sea bacterial isolates (Lauro and Bartlett, 2008). In particular, the *phr* gene was found in other deep‐sea Vibrio (e.g., *V. antiquarius* and *V. diabolicus*; Goudenège *et al*., 2014; Hasan *et al*., 2015) as well as in piezosensitive *Photobacterium profundum* strain 3TCK (Zhang *et al*., 2016). It is suggested that these strains might be in an early stage of their adaptation to the deep biosphere and the vestigial *phr* gene functioning in euphotic bacteria was not yet lost.

### Virulence

*In silico* genome analysis revealed a wide range of features associated with bacterial virulence with a potential role in environmental adaptation. In particular, a vibriolysin metalloprotease showing 94% nucleotide sequence similarity to the metalloprotease Vsm secreted by the oyster pathogen *Vibrio tasmaniensis* LGP32 strain (Le Roux *et al*., 2007) was found in C‐I of Sal10^T^ genome. Vsm is an essential determinant of oyster lethality in extracellular products of *V. tasmaniensis* LGP32 and *Vibrio aestuarianus* (Binesse *et al*., 2008; Labreuche *et al*., 2010). Nevertheless, the main physiological function of bacterial extracellular metalloproteases is to degrade environmental proteins and peptides for bacterial heterotrophic nutrition (Wu and Chen, 2011).

*rtxACHBDE* gene cluster was also annotated within the genome containing a putative RTX toxin in C‐I (encoded by the gene *rtx*A) and an acyltransferase (*rtx*C), the determinant A Ca^2+^ binding protein (*rtx*H) and a putative type‐I secretion system (*rtx*BDE) in C‐II. So far, in the Splendidus clade, the *rtxACHBDE* gene cluster has been only found in *V. splendidus*, although it is present in other *Vibrio* spp. such as *Vibrio vulnificus* and *V. cholerae*. In *V. vulnificus*, MARTX toxin could protect bacteria from predation by amoebae, which would increase bacterial survival outside the host and would explain the fitness of this species in the marine environment (Lee *et al*., 2013). Resistance to protozoan grazing is also a common mechanism fostering coincidental selection of virulence factors in *Vibrio* species (Erken *et al*., 2013; Van der Henst *et al*., 2018). In *V. splendidus*, the MARTX toxin cluster has been associated with virulence in marine invertebrates, especially oysters, by possibly impairing the host innate immune response. In this study, the identified rtxA toxin (7878 bp) showed low sequence similarity, both nucleotide (72%) and aminoacidic (53.96%), with chromosomic regions of distantly related *Vibrionaceae* bacteria. MARTX toxins are a heterogeneous group of toxins, composed of conserved repeat regions, an autoprocessing protease domain and several effector domains that vary through bacterial species or even from different strains (Kim, 2018). Functional analysis and domain prediction of the protein identified two multifunctional‐autoprocessing repeats‐in‐toxin domains, together with several tandem repeat domains, also identified as Cadherin‐like domains. Besides, an actin cross‐linking domain (ACD) was identified, an effector that is responsible for cytoskeleton disruption (Sheahan *et al*., 2004; Figure S3). More data is required to study the phylogeny of the toxin and further analysis on the protein structure would help to fully characterize this new rtxA toxin. Together with the RTX toxin, three different loci of a T1SS secreted agglutinin RTX were identified in C‐I, two of which are phylogenetically related to other species of the Splendidus clade and the third one, with a total length of 20,082 nucleotides, with homology with species of the Splendidus clade (96%) but also with *V. cholerae* (80%) on their nucleotide sequence.

The ancestral virulence trait R5.7, present in all Splendidus clade virulent populations implicated in oyster mortalities (Bruto *et al*., 2018), has been found within Sal10^T^ genome (Figure S3). This gene has been demonstrated to be necessary but not sufficient for virulence and, therefore, it has been suggested that additional virulence determinants might be involved in virulence of Splendidus populations, such as the MARTX toxin (Bruto *et al*., 2018).

Other genes potentially involved in virulence are also present, including type IV pilin (C‐I), *pil*A, which encodes proteins expressed during human infection. Type VI secretion system (T6SS), which is present in Sal10^T^ C‐I, has been demonstrated to be important in virulence being related to anti haemocyte activity in oyster (Rubio *et al*., 2019). A role of T6SSs in environmental fitness of *Vibrio* was also recently suggested (Salomon *et al*., 2013). In contrast, Sal10^T^ does not contain a type III secretion system (T3SS), responsible for the injection of effector proteins into target host cells. Sal10^T^ genome also encodes different haemolysin genes, including a thermolabile haemolysin precursor in C‐II with sequence similarity to that in other Splendidus clade species (*V. crassostreae*, *V. tasmaniensis*, *V. chagassi* and *V. splendidus*) and *V. alginolyticus*. C‐I includes other putative genes that are predicted to encode haemolysins, along with homologues to ToxR and ToxS, a family of regulator which role in virulence has been showed in *V. cholerae*. Aquatic environments contain limited amount of nutrients and it has been proposed that haemolysins might play a role in environmental adaptation of *Vibrio* species by acquiring nutrients through damage to cells of marine organisms (Matz *et al*., 2011).

The genome also contains two prophages of 27.3 Kb in C‐I (52 proteins) and 35.7 Kb in C‐II (46 proteins) respectively. Prophage in C‐I, identified as CTX‐prophage, may be ascribed to Zot‐enconding prophages by the presence of the Zona occludens toxin (*zot*), accessory cholera enterotoxin (*ace*), and other core genes such as RstA phage‐related protein, RstB phage‐related integrase and RstR phage‐related transcriptional repressor. *Zot* and *Ace* toxin gene sequences were similar to that in *V. splendidus* with 94.55% and 92.75% nucleotide sequence similarities respectively, however its function and role in virulence remains untested. Additionally, insertion of another bacteriophage gene was noted with 85.87% identity to bacteriophage f237 of *Vibrio parahaemolyticus*. Although displaying similar genomic structure on the prophage core genes, the identified prophage exceeds the average length of such *Zot*‐encoding prophages, ranging from 5 kb to 10 kb, probably due to the presence of concatamers upstream. These *Zot*‐encoding prophages have been previously found in several non‐cholerae strains (*V. vulnificus, Vibrio maritimus, Vibrio azureus, Vibrio splendidus, Vibrio crassostreae, Vibrio diabolicus* EX25, *Vibrio diazotrophicus* and *Vibrio halioticoli*), including deep‐sea strains (Hasan *et al*., 2015; Castillo *et al*., 2018), pointing out that these elements are widespread in environmental *Vibrio* species. Conversely, prophage in C‐II, identified as a *Vibrio* phage, only contained genes encoding phage core proteins, such as integrase, tail and assembly proteins, lysozyme, head and capsid proteins, scaffolding proteins and terminase, together with unknown proteins.

Overall, most of the virulence related traits here described which are present in the Sal10T genome are likely to play a role in environmental survival of the bacterium suggesting that the marine ecosystem might foster the selection of strains with pathogenic potential. Knowledge of these traits and their ecological drivers is of pivotal importance to fully understand the emergence of virulence in coastal *Vibrio* pathogens.

### Antibiotic resistance

Sequence analysis using the ‘Comprehensive Antibiotic Resistance Database’ (CARD, Alcock *et al*., 2020) identify five different drug resistance classes in the Sal10^T^ genome including resistance to macrolids, quinolones, tetracycline, beta‐lactams and sulphonamides. Phenotypic resistance was confirmed for tetracycline while phenotypic intermediate susceptibility was observed for ciprofloxacin and ampicillin. Resistance to antibiotics of *V. bathopelagicus* sp. nov. support the role of the marine environment as a reservoir of antibiotic resistance genes (ARGs), encompassing resistances to both natural and synthetic antimicrobials, possibly having a natural origin (D'Costa *et al*., 2011). Interestingly, genes associated with resistance to toxic compounds such as copper homeostasis, cobalt‐zinc‐cadmium, arsenic and chromium were also found in Sal10^T^ genome.

### *V. bathopelagicus* sp. nov. interactions with mussel and oyster haemocytes

**Fig. 3:**
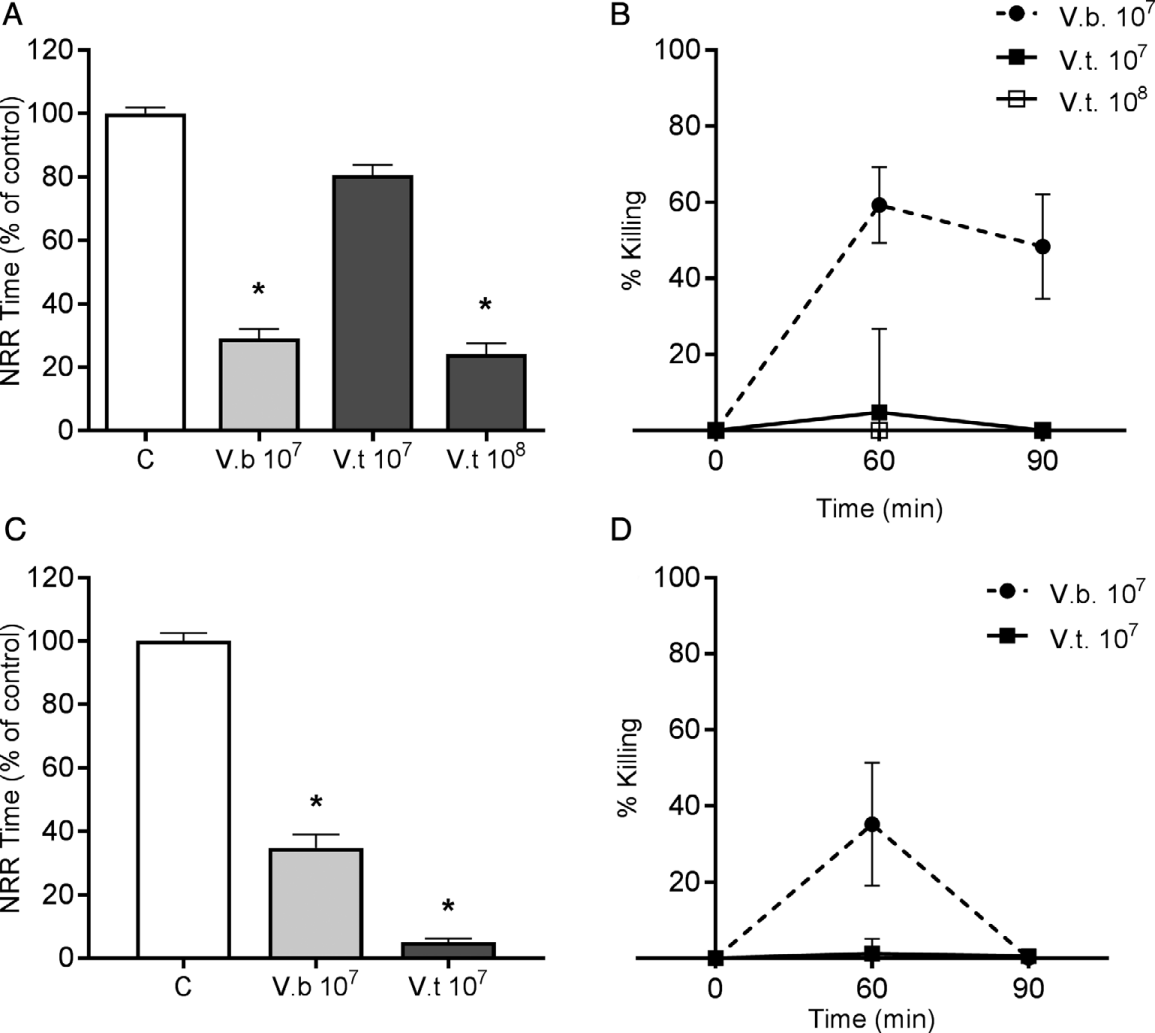
*In vitro* effects of *Vibrio bathopelagicus* sp. nov. on haemocyte lysosomal membrane stability (LMS) and bactericidal activity in the *Mytilus galloprovincialis* (upper panel) and *C. gigas* (lower panel). Upper panel: A. LMS: mussel haemocytes were treated with *V. bathopelagicus* (V.b.) 10^7^ CFU ml^−1^. For comparison, data obtained with *Vibrio tasmaniensis* LGP32 (V.t) at 10^7^ and 10^8^ CFU ml^−1^ are reported; Controls haemocytes (C) were treated with artificial sea water (ASW). B. Bactericidal activity: haemocytes were incubated for different periods of time (60–90 min) with *V. bathopelagicus* sp. nov. at the same concentration utilized in the LMS assay, and the number of viable, cultivable bacteria (CFU) per monolayer was evaluated. Percentages of killing were determined in comparison to values obtained at zero time. Lower panel: oyster haemocytes treated with *V. bathopelagicus* (V.b.) 10^7^ CFU ml^−1^. C. LMS; D. bactericidal activity. Data obtained with *V. tasmaniensis* LGP32 (V.t) at 10^7^ CFU ml^−1^ are also reported. * = *P* < 0.05, Mann–Whitney *U* test.

Potential toxigenicity of *V. bathopelagicus* sp. nov. toward bivalves was investigated in model organisms by *in vitro* challenge of mussel and oyster immune cells (haemocytes). Incubation of *V. bathopelagicus* sp. nov. (10^7^ CFU ml^‐1^) with mussel haemocytes induced a large decrease of lysosomal membrane stability (LMS) a marker of cellular stress (−70% with respect to controls treated with ASW only; Figure 3A). By comparison, the bivalve pathogen *Vibrio tasmaniensis* LGP32 induced a comparable effect only at a concentration ten times higher (10^8^ CFU ml^‐1^). Data on bactericidal activity show that mussel cells retained a significant and sustained ability to kill *V. bathopelagicus* over time (up to 60% of cells were killed after 60 min; Figure 3B).

In oysters the effects of *V. bathopelagicus* sp. nov. on LMS were similar to those observed in mussel haemocytes, however, *V. tasmaniensis* LGP32 caused a stronger lysosomal destabilization (−95% with respect to controls; Figure 3C). Oyster haemocytes showed a lower and transient bactericidal activity against *V. bathopelagicus* sp. nov. and an inability to kill *V. tasmaniensis* LGP32 (Figure 3D). The results obtained for LMS suggest that *V. bathopelagicus* sp. nov. is toxigenic for both mussel and oyster haemocytes. The effects are stronger than those of the bivalve pathogen *V. tasmaniensis* LGP32 in mussels (this work, Balbi *et al*., 2013), and comparable to that of the marine pathogen *V. coralliilyticus* (Balbi *et al*., 2018a). The effects of *V. bathopelagicus* sp. nov. on oyster haemocytes were noticeable as observed with *V. tasmaniensis* LGP32, a known pathogen responsible for oyster mortality. However, despite bacterial recognition and fast induction of cellular responses, the immune response of bivalve haemocytes depends on activation of intra and extracellular pathways leading to both cell‐mediated and humoral effectors, whose activity does not always necessarily lead to successful elimination of pathogens. In this light, mussel haemocytes, despite the initial stress conditions, showed an efficient bactericidal activity towards *V. bathopelagicus* sp. nov. over time, indicating substantial recovery. In contrast, oyster haemocytes display a low ability to overcome the stress conditions induced by *V. bathopelagicus* sp. nov., as shown by the low bactericidal activity. This suggests a higher toxigenicity for oyster compared with mussels. This is a known general trait, with oysters more susceptible to *Vibrio* pathogens and subject to mortality, and mussels rather more resistant to similar infections (Destoumieux‐Garzón *et al*., 2020).

### *V. bathopelagicus* sp. nov. effects on *M. galloprovincialis* larval development

**Fig. 4:**
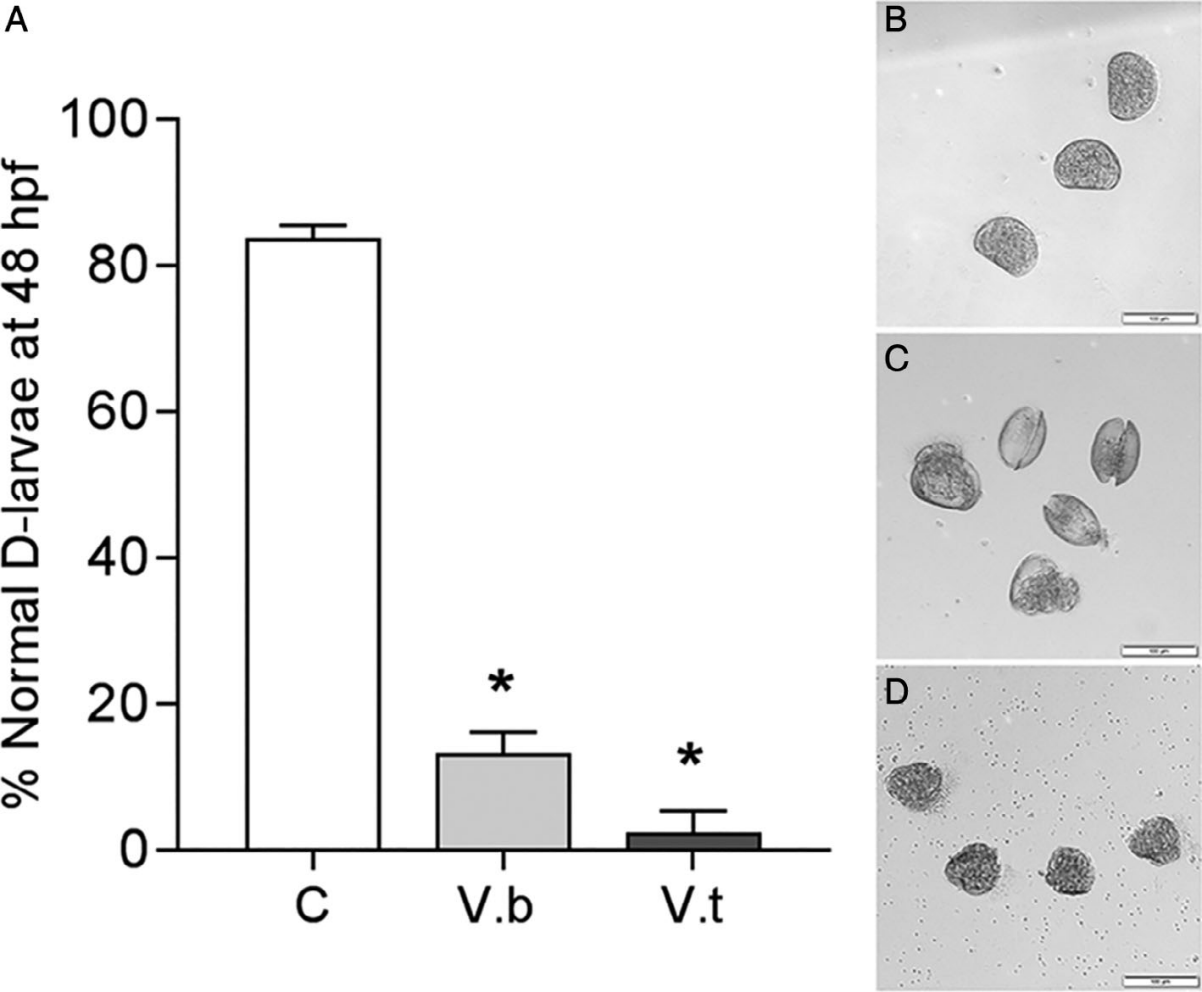
Effects of *Vibrio bathopelagicus sp. nov*. (V.b.) and *Vibrio tasmaniensis* LGP32 (V.t; 10^6^ CFU ml^−1^) on *Mytilus galloprovincialis* larval development in the 48 h embryotoxicity assay. A. Percentage of normal D‐shaped larvae at 48 hpf. Data represent the mean ± SD of four experiments carried out in 96‐multiwell plates (six replicate wells for each sample). * = *P* < 0.01, Mann–Whitney *U* test. B–D. Representative images of control (B) and vibrio*‐*exposed larvae (C,D). Scale bars = 100 μm. B. normal D‐veligers, characterized by regular shells with straight hinge; C. *V. bathopelagicus* induced larval death, as shown by open shells and release of soft tissues. D. *Vibrio tasmaniensis* resulted in arrested development, as shown by the presence of larvae withheld at the trocophora stage.

The possible effects of *V. bathopelagicus* were also evaluated on early larval development of mussels by the standard 48 h embryotoxicity assay, and the results are reported in Figure 4. *V. bathopelagicus* induced a dramatic decrease in the percentage of normal D‐larvae at concentrations as low as 10^6^ CFU ml^−1^ (Figure 4A), indicating that early larval stages of mussels are highly sensitive to the toxicity of this species. A comparable effect was induced by the same concentration of *V. tasmaniensis* LGP32. When the effects of both Vibrios on larval phenotypes were evaluated (see representative images of control and exposed larvae in Figure 4B and C), *V. bathopelagicus* induced larval death (Figure 4C), whereas exposure to *V. tasmaniensis* resulted in arrested larval development (Figure 4C), as previously observed with *V. aestuarianus* and *V. coralliilyticus* (Balbi *et al*., 2019). The results suggest that distinct mechanisms may be involved in the pathogenicity towards mussel larvae of *V. bathopelagicus* sp. nov., with respect to those of other common marine vibrios.

## Conclusions

Virulence is a complex phenotype that requires a susceptible host. In environmental pathogens, defined as microorganisms that usually spend a substantial part of their life cycle outside the host, virulence is believed to arise not only from selection pressures imposed by the interaction with the host, but also from the adaptation of traits that play a more fundamental role for bacterial life outside the host. In marine bacterial pathogens such as vibrios, virulence factors may have an ancestral origin and play a significant role for bacterial life in aquatic habitats.

The deep sea presents peculiar physical–chemical parameters such as high hydrostatic pressure, very low nutrient content and physical separation of water masses that likely preclude significant interaction between deep sea microbes with coastal and shallow‐water marine organisms. Accordingly, environmental adaptation rather than interaction with the bivalve host is likely a primary driver that led to the origin of virulence traits in Sal10^T^ deep‐sea strain. Association with an animal host (e.g., free living protozoa or zooplankton organisms) in the deep‐sea environment cannot be excluded and may also have played a role in coincidental selection of virulence factors responsible of the pathogenicity of this strain towards bivalve molluscs.

Overall, *V. bathopelagicus* Sal10^T^ strain isolated from a remote Mediterranean bathypelagic site, is an example of a planktonic marine bacterium with genotypic and phenotypic traits associated with animal pathogenicity, that might have played an evolutionary role in the origin of coastal marine pathogens.

## Experimental procedures

### Sample collection and Sal10^T^ strain isolation

Sampling was conducted at Sal10 station in the Ionian Sea from the R/V Urania during SALINE2014 (October–November 2014) cruise. Seawater samples were collected from the top of aphotic water column (200 m depth) down to the seabed (30 m above the bottom) using 12‐L Niskin bottles housed on a rosette (General Oceanics, Miami, FL, USA). To measure conductivity, temperature, pressure and oxygen, a calibrated Seabird SBE9/11+CTD was employed.

All 12‐L Niskin bottles were equipped with silicone rubber closure and tubing that had been carefully cleaned and sterilized to avoid introducing contaminants during sampling. Water samples were collected in sterile glass bottles and filtered on 0.2 μm pore size membrane filters with a sterile syringe. The membrane was subsequently transferred in a 50 ml Falcon vial containing alkaline peptone water (APW) culture medium and placed at environmental temperature for 48 h. 100 μl of the resulting solution were then plated on TCBS agar plates and the microbial growth was monitored every 12 h. Sal10^T^ colonies were isolated via stripping, re‐cultured and subsequently transferred in glycerol (10%) and deep frozen (−80°C).

### Genomic DNA isolation and whole genome sequencing

An overnight cell culture of Sal10^T^ strain was prepared for genomic DNA extraction and High Pure PCR Template Preparation Kit (Roche Diagnostics) was used, following manufacturer's protocol, for genomic DNA isolation. DNA concentration and quality were determined fluorimetrically with QuantiFluorTM dsDNA System using a QuantiFluorTM fluorometer (Promega Italia srl, Milano, Italy). The genome of Sal10^T^ strain was sequenced combining short‐read Illumina technology with long‐read MinION sequencing. Illumina sequencing was performed on a MiSeq platform (2 × 250 bp). Libraries were prepared using the NexteraXT library kit following the manufactures instructions, with the following modifications, 2/5th of the recommended reagent volume was used, with 1.4 ng of input DNA. The library amplification parameters were adjusted to: 16 cycles of denaturation at 97°C for 10 s, annealing at 55°C for 30 s and extension at 65°C for 60 s. All other parameters were kept the same. Long reads were obtained on a MinION sequencing device (Oxford Nanopore Technologies) using one‐dimensional (1D) genomic DNA sequencing kit SQK‐LSK109 according to Oxford Nanopore Technologies instructions (Version: GDE_9063_v109_revC_23May2018).

Briefly, purified genomic DNA was repaired with NEBNext FFPE repair mix (New England Biolabs). A NEBNext End repair/dA‐tailing Module was utilized to phosphorylate 5′ ends and add dAMP to the 3′ ends of the repaired DNA. Adapter Mix (Oxford Nanopore Technologies) was ligated to the DNA using NEBNext Quick T4 DNA ligase (New England Biolabs). The DNA was purified with AMPureXP beads (Beckman Coulter, Danvers, MA) following each enzymatic reaction. Purified, adapted DNA was sequenced on an MK1B (MIN‐101B) MinION platform with a FLO‐min 106 (SpotON) R9.4 flow cell using MinKNOW software version 1.7.14 (Oxford Nanopore Technologies). After sequencing, Fast5 files were base‐called using Albacore version 2.1.7 (Oxford Nanopore) on a laptop with a 3.3 GHz Intel Core i7 processor. The genome of *V. lentus* CECT 5110^T^ was also sequenced in this study in order to include this species of the Splendidus clade in the phylogenomic analysis, using the paired‐end chemistry in an Illumina MiSeq 2x250 platform.

### Genome assembly and analysis

Bacterial genome assembly was conducted with Unicycler v0.4.7 pipeline, selecting the bold mode for assembly, by generating a hybrid sequence between short Illumina reads and long MinION reads (Wick *et al*., 2017).

Protein‐encoding sequences (CDS) were annotated using the PATRIC server v3.6.3 (Wattam *et al*., 2017) and general features were searched within the genome together with genes involved in deep‐sea adaptation, in order to predict the biology of Sal10^T^ strain in such environment. Virulence genes were searched by BLASTn analysis with default parameters using the Virulence Factors of Pathogenic Bacteria Database (VFDB; Chen *et al*., 2012), Victors Database (University of Michigan, USA) and PATRIC_VF (Wattam *et al*., 2017). ARGs and heavy metal resistance genes (HMRGs) were searched using the Antibiotic Resistance Database (ARDB; Liu and Pop, 2009) and the CARD (Jia *et al*., 2017). The five mentioned databases are included at the Specialty Genes tool available at the PATRIC server (Wattam *et al*., 2017). Search of specific virulence factors within the genome were performed manually using the Blast service. Additionally, annotation was performed using the Prokka v1.13 software (Seemann, 2014) and Prodigal v2.6.3 (Hyatt *et al*., 2010).

Presence of mobile genetic elements and putative Genomic Islands were predicted on IslandViewer 4 (Bertelli *et al*., 2017) and prophage‐like elements were identified by running each bacterial chromosome in PHASTER (Arndt *et al*., 2016). The detection of secondary metabolite biosynthesis gene clusters was determined using anti‐SMASH 5.0 database (Blin *et al*., 2019).

Protein function and domain prediction was performed using both BLASTP 2.11.0+ (Stephen *et al*., 1997) and InterPro database (Blum *et al*., 2021).

### Phylogenetics and phenotypic characterization

Sequence similarity of 16S rRNA was determined using the EzTaxon‐e server (https://www.ezbiocloud.net; Yoon *et al*., 2017), and the relatedness of Sal10^T^ strain and closely related Splendidus clade species was inferred by the analysis of the 16S rRNA gene sequence and a MLSA based on five housekeeping genes (*atp*A, *pyr*H, *rec*A, *rpo*A, *rpo*D). Genes were aligned using CLUSTALW (Larkin *et al*., 2007) implemented in MEGA X software (Kumar *et al*., 2018). The same software was used for the phylogenetic analysis using both neighbour‐Joining (NJ) and Maximum Likelihood algorithm (Kumar *et al*., 2018), and the bootstrap support for individual nodes was calculated with 1000 replicates. Additionally, a phylogenetic analysis of Sal10^T^ and 11 Splendidus clade species (*V. toranzoniae* CECT 7225^T^, *V. splendidus* DSM 19640^T^, *V. gigantis* LGP13^T^, *V. celticus* CECT 7224^T^, *V. crassostreae* LGP7^T^, *V. atlanticus* CECT 7223^T^, *V. tasmaniensis* LGP32^T^, *V. coraliirubri* Corallo1^T^, *V. kanaloae* CCUG 56968^T^, *V. lentus* CEC 5110^T^ and *V. gallaecicus* DSM 23502^T^) was carried out using the Maximum Likelihood estimation using RAxML (Stamatakis, 2014), with the pipeline implemented in the PATRIC server (Wattam *et al*., 2017), based on 100 housekeeping genes. Besides, genome similarity was calculated using the Average Nucleotide Identity (ANI) and the *in silico* DNA‐DNA hybridization (*is*DDH) indices using OrthoANI (Lee *et al*., 2016) and Genome‐to‐Genome Distance Calculator implementation (Meier‐Kolthoff *et al*., 2013) respectively.

The deep‐sea isolate was subjected to the following phenotypic tests (Mac Faddin, 2006): cell morphology and motility, Gram stain, oxidase, catalase, oxidation/fermentation test, gas and acid production from glucose, indole, methyl red, Voges–Proskauer reaction, utilization of citrate, Thornley's arginine dihydrolase test, Moeller's decarboxylases for arginine, lysine and ornithine, nitrate reduction, hydrolysis of gelatine, Tween 80, amylase and aesculin. Salt tolerance test was performed on Basal medium agar [BMA, neopeptone (4 g L^−1^), yeast extract (1 g L^−1^), bacteriological agar (15 g L^−1^)] supplemented with 1, 2, 3, 4, 5, 6, 7, 8, 9, 10, 12, 15% NaCl and with 0.5 1, 2, 3, 4, 4.5, 5, 6, 7, 8, 9, 10, 12, 15% sea salts. Growth at different temperatures (4, 20, 25, 30, 37 and 44°C) and pH (1–12) were also determined. Phenotypic characterization was complemented by using commercial miniaturized tests API 20NE and API ZYM. The following substrates were tested: ribose, arabinose, xylose, glucose, d‐galactose, maltose, lactose, melibiose, salicin, amygdalin, mannitol, myo‐inositol, glycerol, sodium acetate, propionic acid, citric acid, lactic acid, trans‐aconitic acid, succinic acid, glycine, leucine, serine, threonine, glutamic acid, tyrosine, ornithine, citrulline, lysine, alpha‐ketoglutaric acid, galacturonic acid, glucuronic acid, pyruvic acid, d‐gluconate, phenylacetate, phenylalanine, histamine, sarcosine, 3‐hydroxybutyric acid, *N*‐acetyl‐d‐glucosamine, fumaric acid, malic acid and saccharic acid.

### Antibiotic sensitivity assay

Antimicrobial susceptibility testing (AST) was performed by disk diffusion according to the European Committee on Antimicrobial Susceptibility testing (EUCAST, 2020a). Based on genomic information Sal 10 isolate was tested against a panel of 5 antimicrobial agents using antibiotic disks (Oxoid, UK) on Mueller–Hinton agar (MH; Oxoid, UK). The following agents were included: ciprofloxacin (CIP, 5 μg), chloramphenicol (C, 30 μg), tetracycline (TE, 15 μg), trimethoprim + sulfamethoxazole (SXT 1,25‐23,75 μg), ampicillin (AMP, 10 μg). *Escherichia coli* ATCC 25922 was included as quality control. The isolates were classified as sensitive (S), intermediate susceptible, increased exposure (I) or resistant (R) according to EUCAST breakpoints for Enterobacterales (EUCAST, 2020b). Breakpoints were unavailable for TET and no inhibition zone was the criterion used for classifying the isolate as resistant.

### Animals, haemolymph sampling, haemocyte monolayers

Mussels (*M. galloprovincialis*), 4–5 cm long, were purchased from an aquaculture farm (La Spezia, Italy) in November 2020 and acclimated for 24 h in static tanks containing aerated artificial sea water (ASW), 35 ppt salinity (1 L/mussel) at 18°C. Oysters (*C. gigas*), 8–10 cm long, were purchased from an aquaculture farm (Bretagne, France) at the same time of the year and acclimated in the same conditions at lower salinity (23 ppt). Haemolymph was extracted from the posterior adductor muscle of 4–6 individuals as previously described (Balbi *et al*., 2013, 2018a,b) filtered through a sterile gauze and pooled. Haemocyte monolayers from mussels and oysters were prepared on glass slides as previously described (Balbi *et al*., 2013, 2018a,b).

### Bacterial cultures

*V*. *bathopelagicus* Sal10^T^ and *V. tasmaniensis* LGP32 were cultured in Zobell medium at 20°C and 23°C respectively, under static conditions. After overnight growth, cells were harvested by centrifugation (4500*g*, 10 min), washed three times with ASW, salinity 35 ppt and resuspended to an A600=1 (about 10^9^ CFU ml^−1^). TCBS agar (Scharlau Microbiology, Italy) was used for culturing both strains.

### In vitro challenge of bivalve haemocytes with *V. bathopelagicus sp. nov*.

Haemocyte monolayers were incubated for 30 min at 18°C with suspensions of *V*. *bathopelagicus* Sal10^T^, 10^7^ CFU ml^−1^ in ASW (35 and 23 ppt salinity for mussel and oysters respectively). Control haemocyte samples were run in parallel. LMS was evaluated as a marker of cellular stress by the Neutral Red Retention Time (NRRT) assay as previously described (Balbi *et al*., 2013, Balbi *et al*., 2018a,b). After incubation with bacteria, the medium was removed and cells were incubated with a neutral red (NR) solution in ASW (final concentration 40 μg ml^−1^ from a stock solution of NR 40 mg ml^−1^ in DMSO); after 15 min excess dye was washed out and 20 μl of ASW were added. For oyster haemocytes, ASW at 23 ppt salinity was utilized and assay conditions were optimized (NR final concentration 20 μg ml^−1^, incubation time 7 min). Every 15 min, slides were examined under optical microscope and the percentage of cells showing loss of dye from lysosomes in each field was evaluated. For each time point, 10 fields were randomly observed (8–10 cells each). The end‐point of the assay was defined as the time at which 50% of the cells showed sign of lysosomal leaking, i.e., the cytosol becoming red and the cells rounded. All incubations were carried out at 18°C. For comparison, in mussel, haemocytes parallel experiments were carried out with the bivalve pathogen *V. tasmaniensis* LGP32.

### Bactericidal activity

Bactericidal activity of haemocyte monolayers was evaluated as previously described (Balbi *et al*., 2013, 2018a). Haemocyte monolayers were incubated with *V. bathopelagicus* Sal10^T^ 10^7^ CFU ml^−1^, at 18°C. Immediately after the inoculum (*T* = 0) and after 60 and 90 min of incubation, supernatants were collected and haemocytes were lysed by adding 0.5 ml of filter sterilized ASW added with 0.05% Triton X‐100 and by 10 s agitation. The collected supernatants and haemocyte lysates were pooled and 10‐fold serial diluted in ASW. Diluted samples (10 μl drop) were plated onto TCBS Agar in triplicate. After overnight incubation at 20°C, the number of colony‐forming units (CFU) per millilitre was determined. Percentages of killing were determined in comparison to values obtained at *T* = 0. The number of CFU in control haemocytes never exceeded 0.1% of those enumerated in experimental samples. Same procedure was used for *V. tasmaniensis* LGP32, but diluted samples were plated onto LB agar 3% NaCl.

### Mytilus early larval development assay

The 48 h larval toxicity assay was carried out in 96‐microwell plates as described in Balbi *et al*. (2019). Aliquots of 20 μl of suspensions of *V. bathopelagicus* (obtained from a 10^7^ CFU ml^−1^ stock suspension), suitably diluted in ASW, were added to fertilized eggs in each microwell to reach 10‐fold nominal final concentrations (from 10^1^ to 10^6^ CFU ml^−1^) in a 200 μl volume. For comparison, similar experiments were carried out with *V. tasmaniensis* LGP32. At each dilution step, all suspensions were immediately vortexed prior to use. Microplates were gently stirred for 1 min, and then incubated at 18 ± 1°C for 48 h, with a 16 h:8 h light:dark photoperiod. All the following procedures were carried out following ASTM (2004). Samples were fixed with buffered formalin (4%) and all larvae in each well were examined by optical microscopy using an inverted Olympus IX53 microscope (Olympus, Milano, Italy) at 40X, equipped with a CCD UC30 camera and a digital image acquisition software (cellSens Entry). The acceptability of test results was based on controls for a percentage of normal D‐shell stage larvae > 75% (ASTM, 2004).

### Data analysis

Data are the mean ± SD of at least four independent experiments with each assay performed in triplicate. Statistical analyses were performed by Mann–Whitney *U* test using the GraphPad Prism 5 software.

## Supporting information


**Appendix S1.** Supporting Information.
