# Supplementary material for: A deep‐sea bacterium related to coastal marine pathogens
Source: Environ Microbiol. 2021 Jun 15;23(9):5349–63. doi: 10.1111/1462-2920.15629 (PMC8519021; doi:10.1111/1462-2920.15629)
Supplement: Supplementary file 1 — Appendix S1. Supporting Information. [file EMI-23-5349-s001.pdf]

Supplementary Figure 1. Sampling location and CTD measurement at Sal10^T station in the Ionian Sea

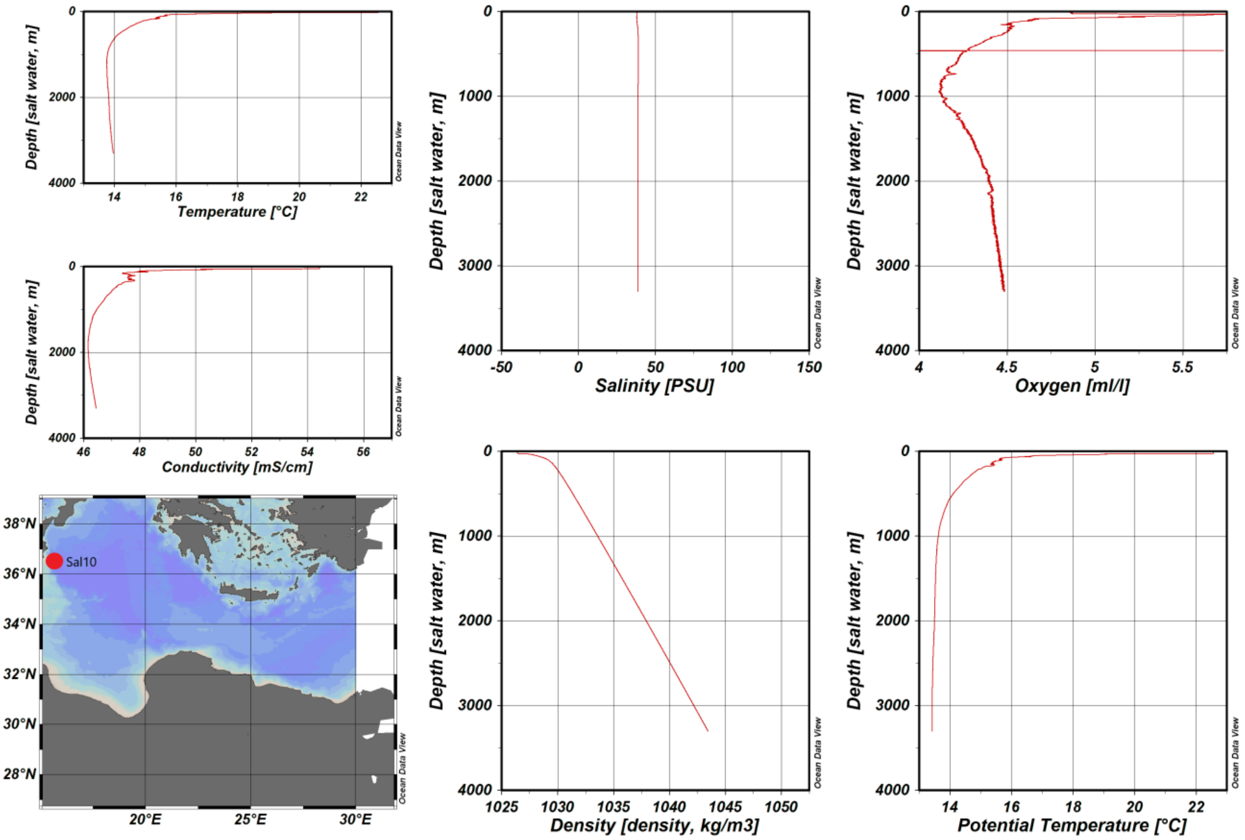

Supplementary Figure 2. RAxML phylogenetic tree based in 100 housekeeping genes obtained from all the Splendidus clade species genomes present in the databases.

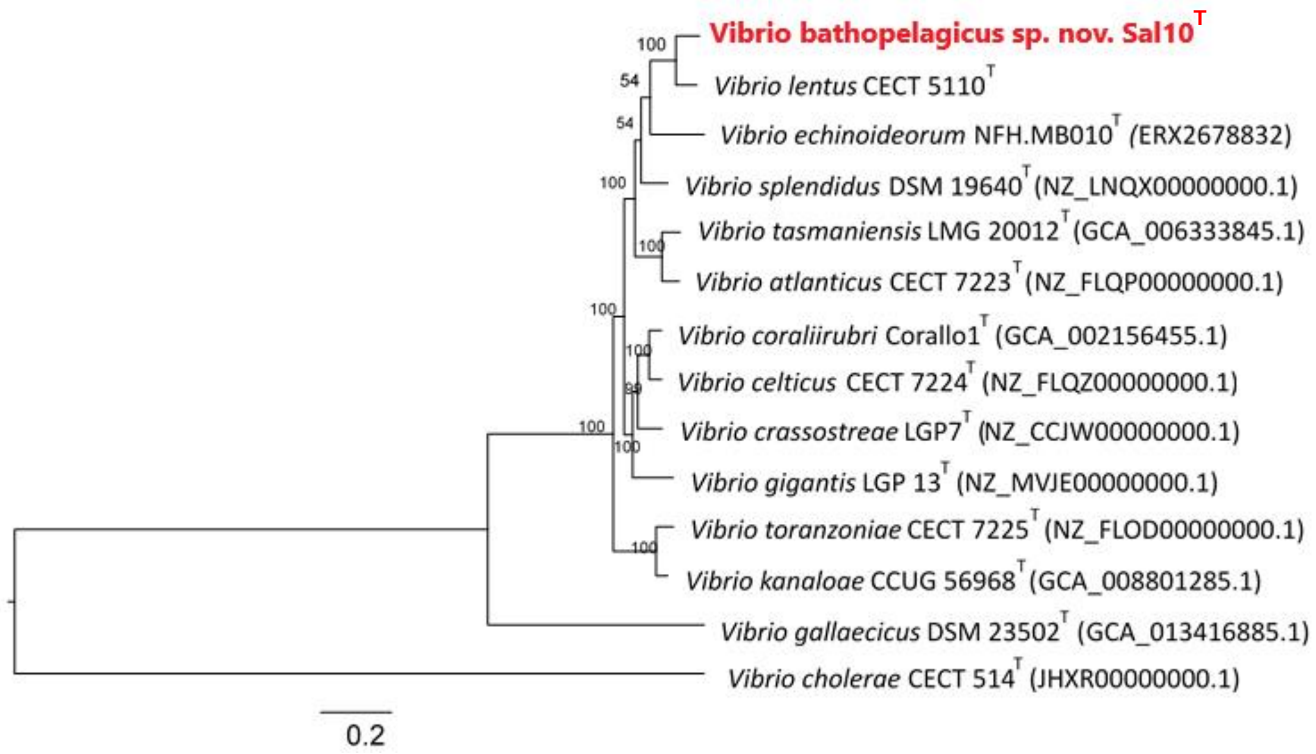

Supplementary Figure 3. Phylogeny of the R5.7 element (A) and functional analysis and domain prediciton of rtxA toxin (B) of V. bathopelagicus sp. nov. Sal10T.

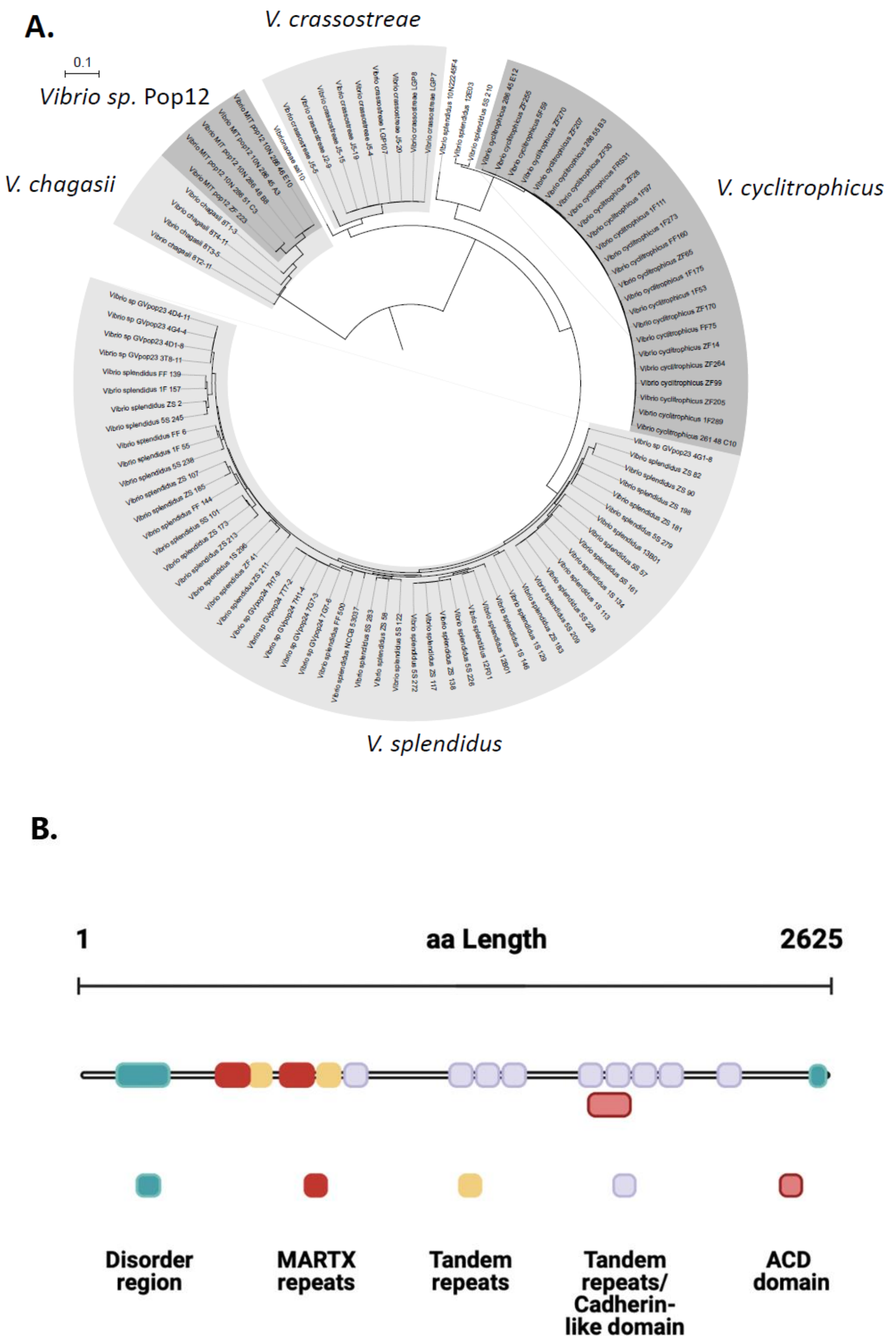

Supplementary Table 1. Distinguishing phenotypic characteristics between Sal10^T strain and closely related *Vibrio* species.

**Test**	***Vibrio bathopelagicus*** **Sal 10^T**	***V. lentus*** **CECT 5110^T**	***V. splendidus*** **ATCC 33125^T**	***V. echinoideorum*** **NFH.MB010^T**
**O/129**	**S**	**R**	**R**	**S**
**ADH**	**-**	**-**	**-**	**+**
**VP**	**-**	**+**	**+**	**-**
**NO_3 reduction**	**+**	**-**	**+**	**+**
**6 %NaCl**	**-**	**+**	**+**	**-**
**Beta-galactosidase**	**-**	**+**	**+**	**+**
**Glucuronic acid**	**+**	**-**	**+**	**-**
**Maltose**	**+**	**-**	**+**	**+**
**Aesculin hydrolysis**	**+**	**-**	**+**	**-**
**D-galactose**	**+**	**-**	**+**	**+**
**Valine arylamidase**	**+**	**+**	**W**	**-**
**Trypsin**	**+**	**W**	**W**	**-**

Supplementary Table 2. Genomic overview of the Sal10^{T} strain analyzed in this study.

	Chromosome I	Chromosome II
**Length (bp)**	3,649,238	2,018,969
**GC content (%)**	44.18	43.92
**CDS**	3,255	1,797
**Proteins with functional assignments**	2,589	1,271
**Hypothetical proteins**	666	526
**tRNA**	118	16
**rRNA**	43	3

Supplementary Table 3. Secondary metabolite biosynthesis gene clusters identified within C-I of $Sal10^T$ strain.

	Description	Number of clusters	Gene	Number of genes
**Cluster 1**	Betalactone	1	HDAPKBCK_00411-HDAPKBCK_00433	23
**Cluster 2**	Arylpolyene	1	HDAPKBCK_00797-HDAPKBCK_00839	43
**Cluster 3**	PUFA	1	HDAPKBCK_00907-HDAPKBCK_00934	28
**Cluster 4**	Heterocyst glycolipid synthase-like PKS	1	HDAPKBCK_00916-HDAPKBCK_00943	28
**Cluster 5**	Bacteriocin	2	HDAPKBCK_01602-HDAPKBCK_01612	11
			HDAPKBCK_03233-HDAPKBCK_03242	10
**Cluster 6**	Non-ribosomal peptide synthetase cluster	1	HDAPKBCK_01724-HDAPKBCK_01765	42
